# Transcultural Validation of the Five-Item Dry Eye Questionnaire for Indonesian Populations

**DOI:** 10.7759/cureus.72288

**Published:** 2024-10-24

**Authors:** Nina A Noor, Damara Andalia, Niluh A Sri Ramandari

**Affiliations:** 1 Ophthalmology, JEC Eye Hospitals and Clinics, Jakarta, IDN

**Keywords:** deq-5, dry eye disease, language adaptation, tear film ocular surface society dry eye workshop, validation

## Abstract

Purpose

Indonesian dry eye (DE) disease (DED) prevalence data remain scarce, and Indonesian validations of the five-item Dry Eye Questionnaire (DEQ-5) are unavailable. We aimed to translate and validate an Indonesian language adaptation of the DEQ-5 (INDO-DEQ-5) for local populations.

Methods

Our observational study involved linguistic validation of the translation, reliability testing, dataset screening, and data collection through ophthalmic examinations and interviews. Outpatient adults with dry eye symptoms and formal DED diagnoses were included. Data were captured at patients' enrolment, at their self-administered INDO-DEQ-5, and at ophthalmic examinations. This data was statistically analyzed for agreement, test-retest reliability, and by a receiver operator characteristic (ROC) curve for dry eye sensitivity and specificity.

Results

Tear breakup time (TBUT) was the most frequently used clinical diagnostic parameter, with 96.8% of DED patients having abnormal TBUT. We captured 87.23% of all true DE cases, while the ROC and area under the ROC curve analyses found similar sensitivity between the INDO-DEQ-5 and the composite clinical diagnosis of DE, and TBUT was equivalent to the INDO-DEQ-5 in clinical diagnoses. The level of reliability of the INDO-DEQ-5 was moderate, and subscales were moderate or substantial.

Conclusion

The prediction of DE with TBUT was similar to predictions using the composite definition of clinical DE diagnosis, while the INDO-DEQ-5 diagnostic accuracy was similar to that of TBUT or a composite clinical diagnosis. Thus, the INDO-DEQ-5 is a reliable tool for the clinical evaluation and diagnosis of DE in Indonesian populations and will enable clinicians to rapidly collect accurate data to improve therapeutic management.

## Introduction

Dry eye (DE) disease (DED) is a common multifactorial disorder of the tear film that progressively damages the ocular surface [1]. While some patients experience mild ocular symptoms such as discomfort, others suffer severe ocular fatigue. These ocular discomfort and visual disturbances [2, 3] can negatively impact health- and vision-related daily activities and quality of life (QoL) [4-6], causing a substantial economic burden of disease [2, 3]. Besides age, other DED risk factors include environmental factors (e.g., frequent use of visual displays, low environmental humidity), female gender [3, 7-9], medical conditions including vitamin deficiencies or comorbidities (e.g., Sjӧgren's syndrome, diabetes, conjunctival disorders, blepharitis, rheumatoid arthritis), and use of specific medications including those for gout, HIV and thyroid disease [3, 10-13].

Differences in study cohorts and a lack of standardization in the clinical definitions for DED diagnosis across population studies [6] have resulted in highly variable prevalence rates, from 4.3% to 75% [8, 9, 14-18]. The widely cited 2017 Dry Eye Workshop (DEWS) II report, which collected estimates worldwide, found a prevalence of 5 to 50% [3]. North American data on individuals 50 years and older (mainly in the United States) indicated a prevalence of 7.8% in women [9] and 4.3% in men [8], while individuals over 18 years old had a 6.8% prevalence [7], and those between 48 and 91 years had a 10-year incidence of 21.6% [11]. Currently, the definition of DED commonly used by ophthalmologic researchers and clinicians is that used by the Tear Film and Ocular Surface DEWS II (TFOS DEWS II) report as a "multifactorial disease of the ocular surface characterized by a loss of homeostasis of the tear film, and accompanied by ocular symptoms in which tear film instability and hyperosmolarity, ocular surface inflammation and damage, and neurosensory abnormalities play etiological roles" [19].

Unfortunately, epidemiological data on DED prevalence remain particularly scarce in Southeast Asia, with limited questionnaire-based reports suggesting a highly variable, 21 to 73% prevalence [3, 8, 9, 14]. We were most interested in the DED prevalence within our geography of practice in Indonesia. One Indonesian province with an average population age of 37 years reported a 27.5% prevalence using population-based data from self-diagnostic questionnaires; however, objective tests were excluded [4]. Other studies have noted that DED affects only 22.5% of Indonesians [20]. As aging increases DED prevalence, most studies in Indonesia have been conducted in older patients, with few studies including younger individuals. In addition, data is also limited on DED prevalence in more elderly Indonesians [20]. Therefore, a gap exists in DED data in both younger and much older Indonesian patients [20]. 

Different methods for evaluating DED exist, including clinical assessments such as tear breakup time (TBUT), osmolarity measurements, ocular surface staining [21], or questionnaires that analyze different aspects of DED symptoms. The Dry Eye Questionnaire 5 (DEQ-5) is unique. In addition to its simplicity with only five questions, it also measures symptoms in four dimensions, is self-administered, and evaluates the frequency and intensity of eye discomfort and dryness, as well as the frequency of eye tearing [22]. The aforementioned variations in global or regional DED incidences (including within Indonesia) were also partly due to the use of different types of questionnaires in studies that screened and graded individuals for DED and DED severity. Validated questionnaires allow symptoms to be tested more objectively and enable the results to be compared between researchers [23]. Of the many available questionnaires [24-27], the DEQ-5 is a validated, widely used instrument for the diagnosis of DE [22, 27-31].

To our knowledge, there are no studies validating the DEQ-5 for the Indonesian population, but the availability of such a tool would potentially enable patients to be stratified according to the severity of their DE symptoms, allow the attending ophthalmologist to assess the suitability of more personalized disease management approaches, and advance our understanding of symptom characteristics in DED for these individuals. Thus, our goal was to adapt the DEQ-5 to the Indonesian language to create the Indonesian DEQ-5 questionnaire (INDO-DEQ-5) and validate it in the Indonesian population.

## Materials and methods

Study design

This hospital-based, cross-sectional, observational study was conducted at two sites (JEC Eye Hospital Kedoya and Menteng) in Indonesia. This study was conducted in three phases: linguistic validation of the questionnaire translation, primary data collection and reliability testing, and screening analysis of the primary dataset. Data were collected from ophthalmic examinations and interviews and were reviewed by all investigators or researchers. The study was conducted according to the Declaration of Helsinki ethical guidelines, and the study protocol was approved by JEC Eye Hospital.

Patients

Included patients were adults aged 16 years or older who presented to the outpatient clinic of JEC Eye Hospital Kedoya due to a chief complaint of DE symptoms and had a formal clinical diagnosis of DED (supplementary methods). The included patients had newly diagnosed or established DED. DE symptoms were defined as symptoms related to DED, usually affecting both eyes, including a stinging, burning, or scratchy sensation, stringy mucus in or around the eyes, sensitivity to light, eye redness, foreign body sensation, difficulty wearing contact lenses, difficulty with night-time driving, watery eyes, blurred vision, or eye fatigue. Patients' written informed consents were obtained prior to the inclusion of their data in our observational analyses. Patients were excluded if they required emergency care, could not follow their treating physicians' instructions, or did not consent to participate. At least 20 participants were planned for reliability testing, and at least 200 participants were planned for the screening analysis.

Data collection

Authorized personnel collected data in case report forms and electronic data capture tools maintained by the study site (JEC Eye Hospital) from patients at enrolment through their self-administered DEQ-5 questionnaires and through the results of their ophthalmic examinations. Captured data included demographic data (in accordance with local data privacy regulations), informed consent, inclusion and exclusion criteria, clinic visit dates, medication histories, and ophthalmic assessment results. As this was a non-interventional study, adverse events were not captured, nor was it our intent to assess morbidity outcomes. However, the occurrence of any adverse events and/or serious adverse events was immediately reported as per reporting guidelines and local health care authority guidance and recorded in our database.

Linguistic validation of the translated questionnaire

In accordance with the Mapi Institute guidelines for linguistic validation, four steps were implemented: forward translation, backward translation, testing, and proofreading [32]. Every step was reviewed by an investigation committee comprising three DE specialists, a general practitioner, and a researcher. The forward translation (English to Indonesian) was performed by a qualified translator who was a native speaker of Indonesian. The backward translation (Indonesian to English) consisted of one qualified translator, a native English speaker who had no knowledge of the original English text and who produced a translation of the Indonesian language version. The committee reviewed and compared the Indonesian and original English versions to establish a consensus Indonesian version and then compared the versions to identify differences between the two. The target Indonesian questionnaire was tested on a group of five study participants in a face-to-face, self-administered manner. An interviewer determined how the participant understood each item in the questionnaire and asked for alternative wording for problematic items. The revised questionnaire underwent two rounds of proofreading by the committee.

Reliability testing

The validated DEQ-5 questionnaire was administered on day 0 to a convenience sample of 20 individuals (healthcare workers and staff) who were born and raised in Indonesia and who completed it twice (a second time three days after the first time) to evaluate its intra-test reliability.

Screening analysis

The sensitivity and specificity of the translated Indonesian questionnaire were estimated in the 200 consecutively selected patients with DE symptoms. The participants received a standardized examination TBUT (4 µL of sterile fluorescein placed in the conjunctival sac), fluorescein staining (classified using the Oxford scale [1], and Schirmer's test with anesthesia [33]. DED was considered to be present if one or more of the following positive signs were present: TBUT ≤5s, corneal staining score ≥2, and Schirmer test score ≤5mm in either eye. Meibomian gland dysfunction was considered present if the meibum quality was ≥2 in either eye (0: clear; 1: cloudy; 2: granular; 3: toothpaste type; and 4: no meibum extracted). The cutoff points for the screening criteria were DEQ-5 ≥6 for mild DE symptoms and DEQ-5 ≥12 for severe symptoms.

Statistical analyses

The data were compiled and analyzed using Stata version 15.1 (StataCorp LLC, College Station, Texas) to obtain the Cohen's Kappa test of agreement [34] since there was no rater effect, and results were classified as published previously [35]. Kappa was calculated for all five subscales, and the total score (total of six assessments) was categorized as slight (<0.2), fair (0.2-0.4), moderate (0.4-0.6), substantial (0.6-0.8), or almost perfect (>0.8). The strength of the percentage agreement was interpreted using published guidelines [35]. The test-retest reliability of the translated DEQ-5 questionnaire was confirmed since by repeating the DEQ-5 in the same patients/participants across two time-points, it was the variation of the measurement of the translated instrument (DEQ-5) that was being assessed. Receiver operating characteristic (ROC) analyses were used to examine the sensitivity and specificity of the translated Indonesian language DEQ-5 toward signs of DE. A true variable or gold-standard diagnosis for DE was created using the clinical examination rule that such a patient would have one or more of the following positive DE signs in either eye: TBUT ≤5s, Oxford score (corneal staining score) ≥2, and/or Schirmer score ≤5 mm.

## Results

Participant demographics

We enrolled 200 patients with a median age of 49.7 years (range: 18.3 - 85.7 years), with 142 females, 51 males, and seven individuals of undefined gender (Table 1). 

**Table 1:** Participant demographics and clinical characteristics SD - standard deviation; n - number; TBUT - tear breakup time; DE - dry eye; DEQ - Dry Eye Questionnaire

Dry eye diagnosis in study participants (n=200)	N (%)
Age, mean (SD)	49.7 (16.)
Sex, n (%)	193 (96.5%)
Female	142 (73.6)
Male	51 (26.4)
Clinical diagnosis of DE using any of the following: TBUT, Oxford score, Schirmer score	188 (94%)
TBUT diagnosis of DE	182 (91%)
Oxford score diagnosis of DE	37 (18.5%)
Schirmer score diagnosis of DE	104 (52%)
Moderate DE (DEQ≥6)	87.5%
Severe DE (DEQ≥12)	50%

**Figure 1:**
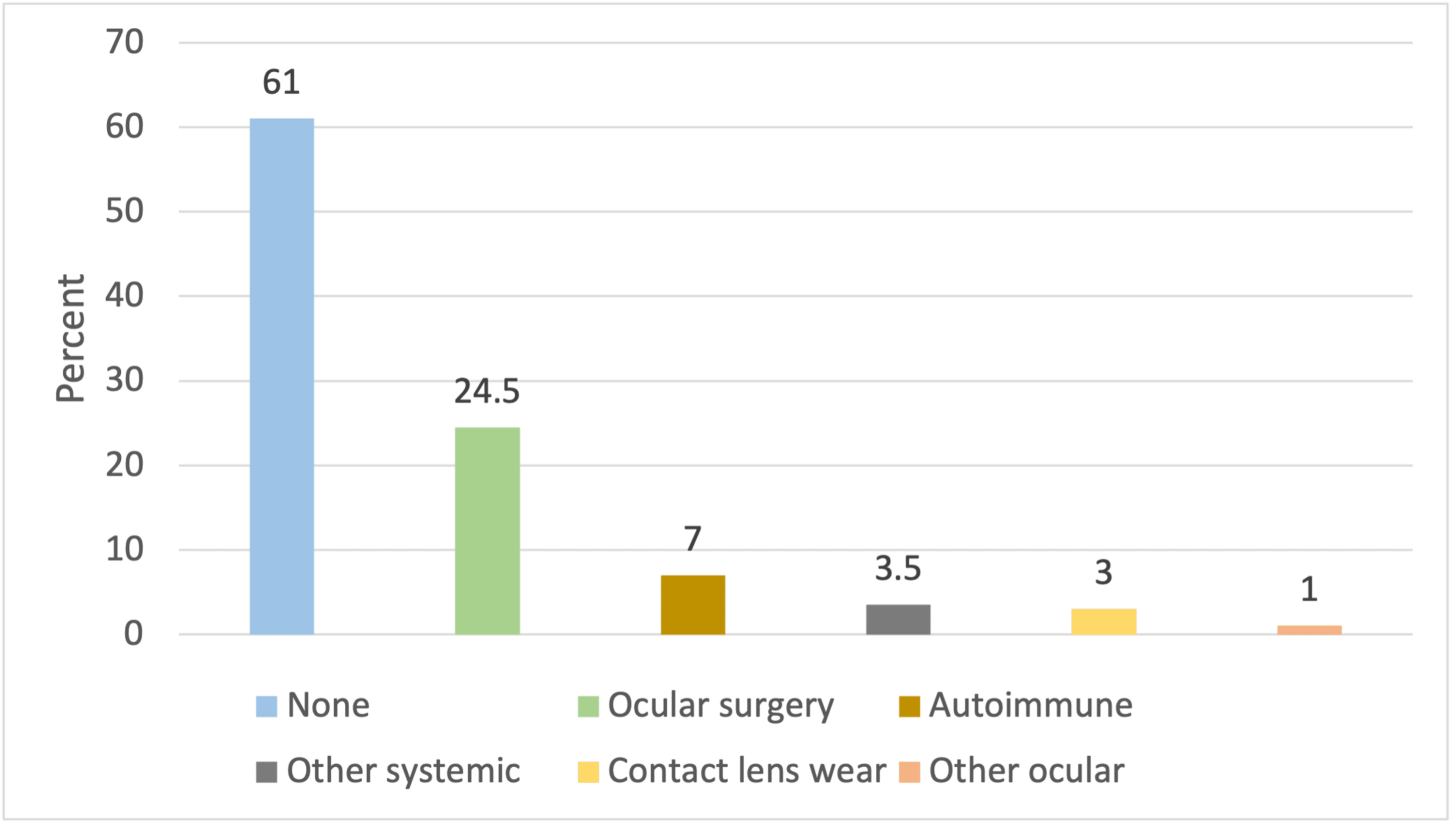
Comorbidities among study participants (n=200); the percentage of patients (%) with the most commonly detected comorbidities is depicted

Of the three clinical indicators (TBUT, Oxford score, and Schirmer Testtscore) of DED, TBUT abnormality was the most frequently used, with 182 of 188 (96.8%) patients with a clinical diagnosis of DE also having a TBUT abnormality. Among the 200 participants, 39% reported comorbidities. The most common comorbidities were ocular surgery (n=49; 24.5%), contact lens wear (n=6; 3%), autoimmune conditions (n=14; 7%) including Sjögren’s syndrome, other systemic diseases (n=7; 3.5%) including thyroid disease, and other ocular diseases (n=2; 1%) including blepharospasm (Figure 1).

Linguistic validation of the translated questionnaire

**Figure 2:**
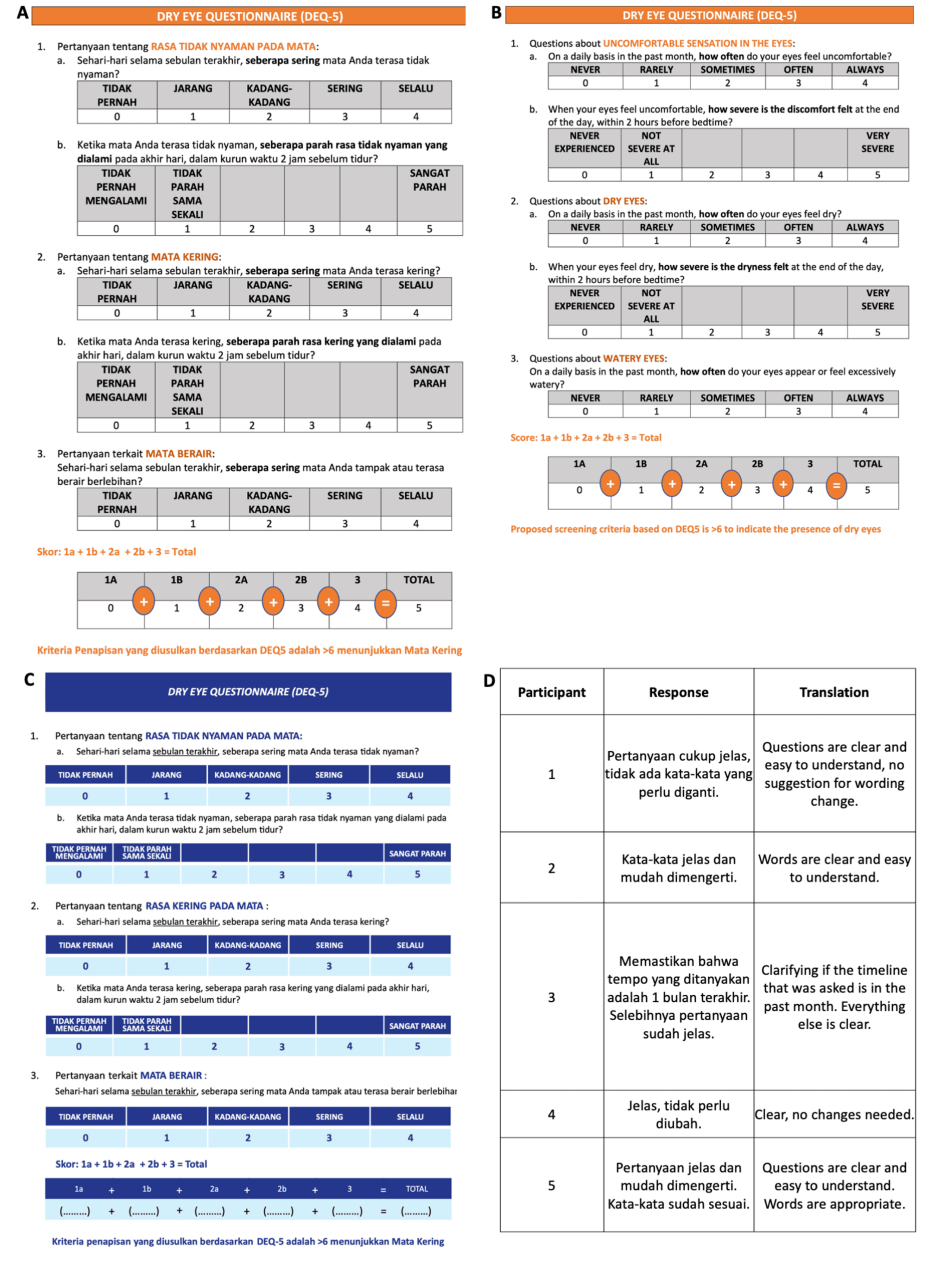
Dry Eye Questionnaire (DEQ-5) translations and in-person testing Forward translation (A) into Indonesian and backward translation (B) into English languages are shown with the consensus version. C is identical to the Indonesian version or the INDO-DEQ-5. Five participants also indicated if they understood the questions (D)

The DEQ-5 questionnaire was forward translated into the Indonesian language (Figure 2A), backward translated back into the English language (Figure 2B), and the versions were compared to identify differences (Figure 2C). This was conducted through discussions and resulted in a single minor change to question two for clarity. Thus, the content of the consensus version in the Indonesian language (i.e., the INDO-DEQ-5) was identical to the English language DEQ-5. For face-to-face testing of the INDO-DEQ-5, five study participants also stated that they clearly understood the questions and did not suggest alternative wordings (Figure 2D). 

Sensitivity-specificity analyses

We considered the sensitivity of DE diagnosis to be more important than the specificity (chance of true positives) of the diagnosis. In other words, when using the translated INDO-DEQ-5 as a diagnostic tool for DE, we were more interested in capturing all true-positive cases, even if this increased the chance of not capturing all disease-free patients. As such, and compared to the composite clinical diagnosis, the threshold (cut-off score) for sensitivity and specificity of the INDO-DEQ-5 for DE diagnosis was set at six (Table 2).

**Table 2:** Sensitivity-specificity analyses for the composite clinical diagnosis versus the translated DEQ-5; a higher LR+ improves the predictability of patients without DE LR- - negative likelihood ratio; LR+ - positive likelihood ratio; DE - dry eye

Cutpoint	Sensitivity (%)	Specificity	Correctly classified	LR+	LR-
≥0	100.00	0.00	94.00	1.000	-
≥2	98.40	0.00	92.50	0.984	-
≥3	96.81	0.00	91.00	0.968	-
≥4	95.74	0.00	90.00	0.957	-
≥5	93.62	0.00	88.00	0.936	-
≥6	87.23	8.33	82.50	0.952	1.532
≥7	84.04	8.33	79.50	0.917	1.915
≥8	79.26	33.33	76.50	1.189	0.622
≥9	71.81	33.33	69.50	1.077	0.846
≥10	63.30	33.33	61.50	0.950	1.101
≥11	57.45	41.67	56.50	0.985	1.021
≥12	49.47	41.67	49.00	0.848	1.213
(≥13	38.30	50.00	39.00	0.766	1.234
≥14	28.72	75.00	31.50	1.149	0.950
≥15	18.62	83.33	22.50	1.117	0.977
≥16	10.11	91.67	15.00	1.213	0.981
≥17	5.85	100.00	11.50	-	0.942
≥18	3.19	100.00	9.00	-	0.968
≥19	2.13	100.00	8.00	-	0.979
≥20	1.06	100.00	7.00	-	0.989
≥21	0.53	100.00	6.50	-	0.995
>21	0.00	100.00	6.00	-	1.000

At this cut-off score, there chance of catching all true DE cases was 87.23%, with an 8.33% chance of catching those without DE. In other words, 82.5% of the study population was correctly classified (Table 2). Additionally, a contingency table analysis comparing the clinical diagnosis of DE to scores from the translated DEQ-5 questionnaire found that the diagnostic methods started to align (correlate) at a score of five (with a score of zero indicating no clinical diagnosis of DE and a score of one indicating a positive clinical diagnosis of DE).

**Figure 3:**
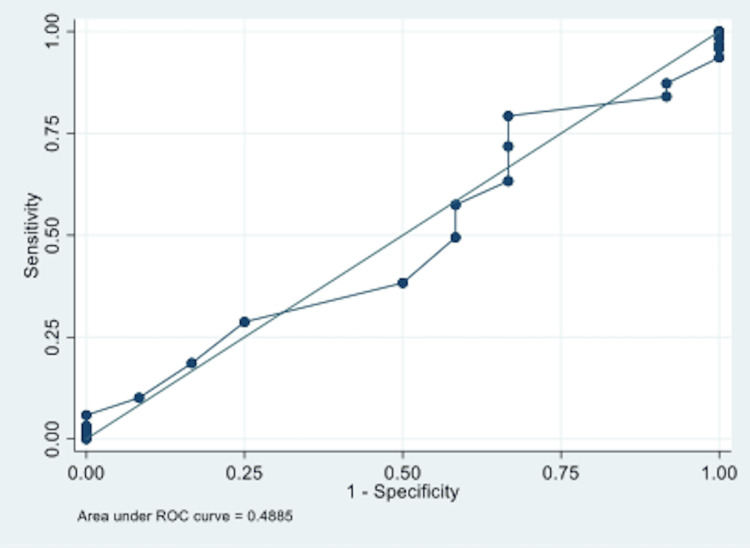
ROC curve for INDO-DEQ-5 compared to clinical diagnosis of dry eye AUC = 0.4910 (95%CI 0.339-0.643) ROC - receiver operator characteristic; INDO-DEQ-5 - Indonesian-language adaptation of the Dry Eye Questionnaire-5; AUC - area under the ROC curve

**Figure 4:**
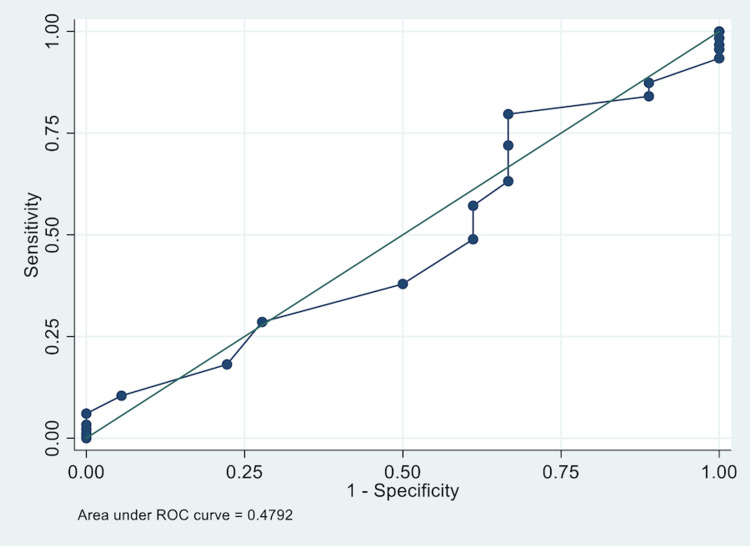
ROC curve for INDO-DEQ-5 compared to TBUT diagnosis of dry eye AUC = 0.4792 (95%CI 0.336-0.622) ROC - receiver operator characteristic; INDO-DEQ-5 - Indonesian-language adaptation of the Dry Eye Questionnaire-5; TBUT - tear breakup time; AUC - area under ROC curve

The ROC and area under the ROC curve (AUC; AUC=0.4910, Figure 3) were calculated for the INDO-DEQ-5 versus the composite clinical diagnosis of DE. At a threshold of six, the DE measurement methods were similar in sensitivity (i.e., one was not more sensitive or specific than the other). We also calculated the ROC/AUC or the sensitivity of TBUT as the gold standard for the clinical diagnosis of DE and found this to be equivalent to the INDO-DEQ-5 (Figure 4).

Together, both sets of ROC/AUC results demonstrate congruence of the INDO-DEQ-5 with the most commonly used clinical tests (TBUT and composite clinical diagnosis of DE). Moreover, another contingency table analysis (Table 3) found that when TBUT was the gold standard for a clinical diagnosis of DE, the sensitivity and specificity of the INDO-DEQ-5 was similar to that when using the composite clinical diagnosis where a cut-off score of 6 could be used (87.36% sensitivity and 11.11% specificity).

**Table 3:** Sensitivity-specificity analyses for TBUT versus the INDO-DEQ-5 TBUT - tear breakup time; INDO-DEQ-5 - Indonesian-language adaptation of the Dry Eye Questionnaire-5; LR- - negative likelihood ratio; LR+ - positive likelihood ratio

Cutpoint	Sensitivity (%)	Specificity	Correctly Classified	LR+	LR-
≥0	100	0	91	1	-
≥2-	98.35	0	89.5	0.9835	-
≥3	96.7	0	88	0.967	-
≥4	95.6	0	87	0.956	-
≥5	93.41	0	85	0.9341	-
≥6	87.36	11.11	80.5	0.9828	1.1374
≥7	84.07	11.11	77.5	0.9457	1.4341
≥8	79.67	33.33	75.5	1.1951	0.6099
≥9	71.98	33.33	68.5	1.0797	0.8407
≥10	63.19	33.33	60.5	0.9478	1.1044
≥11	57.14	38.89	55.5	0.9351	1.102
≥12	48.9	38.89	48	0.8002	1.314
≥13	37.91	50	39	0.7582	1.2418
≥14	28.57	72.22	32.5	1.0286	0.989
≥15	18.13	77.78	23.5	0.8159	1.0526
≥16	10.44	94.44	18	1.8791	0.9483
≥17	6. 04	100	14.5	-	0.9396
≥18	3.3	100	12	-	0.967
≥19	2.2	100	11	-	0.978
≥20	1.1	100	10	-	0.989
≥21	0.55	100	9.5	-	0.9945
>21	0	100	9	-	1

Test-retest reliability. Using Cohen's Kappa test of agreement for reliability and in accordance with those previously published [36], we found the level of reliability of the total score of the INDO-DEQ-5 to be moderate, and that of the subscales to be either moderate or substantial (Table 4) [35]. The variation of results at day zero and at three days post-initiation was fairly consistent. Thus, the translated DEQ-5 survey was reliable. 

**Table 4:** Cohen's Kappa test of reliability of INDO-DEQ-5 Cohen's Kappa scale: slight <0.2; fair 0.2-0.4; moderate 0.4-0.6; substantial 0.6-0.8; almost perfect >0.8 SE - standard error; INDO-DEQ-5 - Indonesian-language adaptation of the Dry Eye Questionnaire-5

	Agreement	Карра	SE	z-value	p-value	Interpretation, level of agreement, or reliability
Total score	45%	0.4	0.0713	5.38	0.000	Moderate
Score 1a	75%	0. 7	0.1301	5.06	0.000	Substantial
Score 1b	75%	0. 7	0.1269	5.25	0.000	Substantial
Score 2a	80%	0.7	0.1308	5.55	0.000	Substantial
Score 2b	80%	0.7	0.1253	5.85	0.000	Substantial
Score 3	65%	0.5	0.1452	3.38	0.0003	Moderate

## Discussion

This study aimed to validate the INDO-DEQ-5 and present its reliability in an Indonesian population. We found TBUT to be the most common clinical indicator of DE (n=182) among our patients (n=200), and its prediction of DE was similar to that when using the composite definition of clinical diagnosis of DE (TBUT, Oxford score, and Schirmer score; n=188, 94%). Moreover, the diagnostic accuracy of the INDO-DEQ-5 was similar (ROC 0.47) to that of the TBUT or to that of a composite clinical diagnosis (ROC. 0.49), whereas Cohen's Kappa found a fair level of reliability for the INDO-DEQ-5, with fairly consistent results at days zero and three. Taken together, our results indicate that the INDO-DEQ-5 survey is a reliable tool for the clinical evaluation and diagnosis of DE in the Indonesian population.

The DEQ-5 was designed to expedite DED diagnosis as it is shorter than other widely used questionnaires, such as the Ocular Surface Disease Index or Ocular Comfort Index, and avoids the need for instruments that may not be easily accessible in resource-limited settings and populations [22, 37, 38]. Through five categories, the DEQ-5 effectively scores the frequency of ocular discomfort, tearing, and dryness [22] experienced by patients. Screening with the DEQ-5 is also recommended [37] as a criterion for DED diagnosis because it can distinguish between different DED severities [22, 37], including the presence or absence of DED or Sjögren's syndrome keratoconjunctivitis sicca, and between cohorts whose self-assessed DED severities varied.

We considered that the sensitivity of DE diagnosis was a more important metric than the specificity (or the chance of true positives) of DE diagnosis. In other words, in using the INDO-DEQ-5 as a diagnostic tool for DE, we were more interested in capturing all true positive cases, even if there was a risk of not catching all disease-free cases. As such, we identified a cutoff point of six in the INDO-DEQ-5 as the likely threshold for DE diagnosis. At this threshold or cutoff point, there is an 87.23% chance of capturing all true DE cases and an 8.33% of capturing patients without DE (Table 2). Clinically, it is also rational to set a higher cutoff point, as the goal is a higher test sensitivity to capture as many potential patients with DED as possible. Thus, within any cohort of Indonesian patients suspected of DE, doctors using this questionnaire should expect to diagnose DED in ~87% of their patient population if their INDO-DEQ-5 score is above six and a lower chance (8.33%) of catching those that do not have DE. As such, with a lower chance of diagnosing true negatives, there is a risk of identifying false positives. However, in clinical practice, this is a safer risk to have than missing true cases, and compromising the ability to catch almost 90% of true cases.

We previously investigated the elderly Indonesian population to determine the prevalence of DED both preoperatively and in terms of its subtypes [39]. A validated DEQ-5 survey for Indonesian language speakers is needed to help improve DED diagnosis, as such tools are difficult to implement given Indonesia's diversity in patient types and healthcare resource accessibility. Outside Indonesia, a DED prevalence between 9% and 30% has been noted when assessment is conducted according to symptoms and signs, or 7% to 52% when assessment is conducted according to symptoms only [3, 40]. As this link between symptoms and signs differs between individuals and DED subtypes [41], more accurate and relevant diagnostic tools are needed to improve DED assessment, ocular surface monitoring, and tracking of DED progression or treatment responses. Such tools are particularly required by general ophthalmologists outside urban areas in Indonesia, who may lack access to specialist tests or instruments or in practices reliant on the capture of symptoms through patient case histories [42, 43]. As symptom quantification closely reflects the signs of DED, validated symptom questionnaires are a reliable clinical tool.

However, a wide variety of diagnostic criteria, study cohorts, and tests (both objective and based on subjective-symptom questionnaires) are used worldwide [37, 38, 42, 44, 45], complicating DED research. Even among cohorts not requiring translation, the DEQ-5 can be difficult to use, as patients cannot differentiate between their eyes feeling grittiness, pain, or soreness, or determine if their poor vision stemmed from blurred vision, watching television, working on computers or being in low-humidity and air-conditioned environments [39]. A culturally adapted, reliable, consistent, and validated screening tool is thus needed for DED in Indonesian populations [38]. To our knowledge, our DEQ-5 survey translation and validation among a hospital-based population with DE are the first to be conducted in Indonesia.

Moreover, given our findings, we believe that ophthalmologists practicing in other countries with similar geographic or demographic variations should also implement their own cultural and linguistic adaptations of the DEQ-5. The INDO-DEQ-5 questionnaire has proven to be both effective and useful but will be further improved by local adaptations. As noted by others [46], one advantage of translating an existing questionnaire into a target language and validating it is that it reduces the time and resource usage associated with developing a new questionnaire and facilitates comparisons between different populations [47]. Our study adds to the limited, peer-reviewed literature on the translation and validation of the DEQ-5 for local, non-English language populations. These include studies from Brazil [46] with Portuguese translations, Chile [28] and Mexico [27] with localized Spanish translations, and most recently, Thailand's Thai language version [21]. One Portuguese translation of the DEQ-5 [47] evaluated the severity and frequency of ocular discomfort, dryness, and tearing in a Brazilian cohort and found its adaptation to be a reproducible and reliable tool, with responses having high internal consistency and correlation. Similar to our study, the Brazilian investigators used two independent translators for each language translation, which provided them with further comparisons, discussions, and the ability to resolve discrepancies for a more accurate linguistic adaptation. In a Chilean study [28], the original DEQ-5 was forward and backward translated into a cultural and Spanish language adaptation, and a psychometric evaluation of the adapted questionnaire's reliability and validity was also included. The median DEQ-5 score recorded by this adaptation was 13 points, with a Cronbach's alpha of 0.8085, indicating good adaptation. Importantly, the psychometric evaluation allowed the investigators to better understand DED symptomology among their patients and how individuals with DED communicate their experiences of these symptoms. In the Thai language adaptation study, 291 patients completed a translated survey to allow a reliability coefficient to be calculated. The Thai adaptation received a Cronbach's alpha of 0.86 and mean item objective congruence, content validity index (CVI), and total-scale CVI values of 0.99, 0.90, and 0.88, respectively. These included users of contact lenses (15%) and eyeglasses (37%), individuals with underlying diseases (<50%), and those currently receiving oral medications or artificial teardrops. This study found the Thai DEQ-5 adaptation to have high validity and reliability and concluded that it could be used for the clinical diagnosis of DED.

## Conclusions

Data on the current prevalence of DED across Indonesia's many regions remain incompletely determined, but this accurate and reliable translation of the DEQ-5 tool will enable more clinicians, particularly those lacking access to sophisticated instruments, to rapidly collect more accurate clinical and epidemiological information about their patient populations, to improve DED therapeutic management and disease understanding. 

## Appendices

Supplementary methods

Dry eye diagnostic procedures

Each subject completed the history taking before undergoing the dry eye clinical tests.

Clinical tests for dry eye disease included the tear-film breakup time test (TBUT), fluorescein staining of the cornea and conjunctiva, and the Schirmer test. TBUT was performed before the other dry-eye tests to avoid any untoward interference. TBUT was measured by wetting a 2% sodium fluorescein 1 mm x 10 mm impregnated strip (OPTITECH Eye Care, India) with 5 μL normal saline and touching this to the surface of the tarsal conjunctival. Three consecutive measurements of the time after a blink to the appearance of the first dark spot were taken for every participant with a stopwatch and the mean was recorded. Ocular surface staining was observed through a slit-lamp 2.5-3.0 minutes after instillation of 2% fluorescein solution with a cobalt blue illumination and an additional yellow filter to enhance the visibility of any fluorescent staining. Fluorescein staining of the cornea and conjunctiva was recorded and graded according to the Oxford ocular surface staining system.

Tear production was examined in both eyes simultaneously with a five-minute Schirmer test using pre-calibrated, sterile, 5×35 mm filter strips (Color Bar; Eagle Vision, Inc., Memphis, TN) without anesthesia. The Schirmer test was performed last so that ocular irritation by the test strip would not interfere with other examination results. The strip was placed temporally in each lower fornix and left in place for five minutes. The participant was suggested to close his or her eyes during the examination. After five minutes, the strip was removed, and the amount of wetting (in millimeters) was recorded from the pre-calibrated strip.
